# Dysmetabolic Circulating Tumor Cells Are Prognostic in Metastatic Breast Cancer

**DOI:** 10.3390/cancers12041005

**Published:** 2020-04-19

**Authors:** Giulia Brisotto, Eva Biscontin, Elisabetta Rossi, Michela Bulfoni, Aigars Piruska, Simon Spazzapan, Cristina Poggiana, Riccardo Vidotto, Agostino Steffan, Alfonso Colombatti, Wilhelm T. S. Huck, Daniela Cesselli, Rita Zamarchi, Matteo Turetta, Fabio Del Ben

**Affiliations:** 1 Immunopathology and Cancer Biomarkers, Centro di Riferimento Oncologico di Aviano (CRO) IRCCS, 33081 Aviano, Italy; giulia.brisotto@cro.it (G.B.); eva.biscontin@gmail.com (E.B.); asteffan@cro.it (A.S.); acolombatti@cro.it (A.C.); matteo.turetta@cro.it (M.T.); fabio.delben@cro.it (F.D.B.); 2 Department of Surgery, Oncology and Gastroenterology, University of Padova, 35128 Padova, Italy; elisabetta.rossi@unipd.it; 3 Veneto Institute of Oncology IOV-IRCCS, 35128 Padova, Italy; cristinapoggiana@gmail.com (C.P.); riccardo.vidotto@outlook.com (R.V.); 4 Department of Pathology, ASUIUD University Hospital, 33100 Udine, Italy; michela.bulfoni@uniud.it; 5 Institute for Molecules and Materials, Radboud University, 6525AJ Nijmegen, The Netherlands; a.piruska@science.ru.nl (A.P.); w.huck@science.ru.nl (W.T.S.H.); 6 Department of Medical Oncology, Centro di Riferimento Oncologico di Aviano (CRO) IRCCS, 33081 Aviano, Italy; spazzapan@cro.it; 7 Department of Medicine (DAME), University of Udine, 33100, Italy; daniela.cesselli@uniud.it

**Keywords:** breast cancer, circulating tumor cells, liquid biopsy, metabolism, droplet microfluidics, pH

## Abstract

Circulating tumor cells (CTCs) belong to a heterogeneous pool of rare cells, and a unequivocal phenotypic definition of CTC is lacking. Here, we present a definition of metabolically-altered CTC (MBA-CTCs) as CD45-negative cells with an increased extracellular acidification rate, detected with a single-cell droplet microfluidic technique. We tested the prognostic value of MBA-CTCs in 31 metastatic breast cancer patients before starting a new systemic therapy (T0) and 3–4 weeks after (T1), comparing results with a parallel FDA-approved CellSearch (CS) approach. An increased level of MBA-CTCs was associated with: i) a shorter median PFS pre-therapy (123 days vs. 306; *p* < 0.0001) and during therapy (139 vs. 266 days; *p* = 0.0009); ii) a worse OS pre-therapy (*p* = 0.0003, 82% survival vs. 20%) and during therapy (*p* = 0.0301, 67% survival vs. 38%); iii) good agreement with therapy response (kappa = 0.685). The trend of MBA-CTCs over time (combining data at T0 and T1) added information with respect to separate evaluation of T0 and T1. The combined results of the two assays (MBA and CS) increased stratification accuracy, while correlation between MBA and CS was not significant, suggesting that the two assays are detecting different CTC subsets. In conclusion, this study suggests that MBA allows detection of both EpCAM-negative and EpCAM-positive, viable and label-free CTCs, which provide clinical information apparently equivalent and complementary to CS. A further validation of proposed method and cut-offs is needed in a larger, separate study.

## 1. Introduction

Breast cancer is the most common cancer among women worldwide, with an estimated 2 million new cases annually [1]. Despite diagnostic and treatment advances, the normal natural progression of the disease is metastatic spread which is the main cause of death [1]. The provision of effective biomarkers to stratify the prognosis or recurrence risk, monitor the treatment response, quantify the minimal residual disease, predict therapy susceptibility and promptly detect the appearance of therapy resistance, is a strong clinical need [2].

Circulating tumor cells (CTCs) are cancer cells spread from primary tumor or metastatic sites and located in body fluids. Their analysis has gained attention as one of the most promising approaches to advance clinical management of cancer, as CTCs constitute tumor material that can be accessed multiple times with minimal invasiveness, providing real-time information on the evolving biology of cancer [3,4,5,6,7]. There is evidence that CTCs can contribute to form distant metastasis [8], and several studies demonstrated that the number of CTCs independently correlates with progression of disease in metastatic breast cancer (mBC) patients, outperforming both serum biomarkers [9,10] and radiologic imaging techniques [11].

The only currently FDA-approved technology for CTC enumeration in mBC is the CellSearch platform (Menarini Silicon Biosystems, Bologna, Italy) that detects CTCs relying on the expression of the epithelial marker EpCAM on their surface. This technology has widely demonstrated the prognostic value of both the number [12,13,14,15] and number variation [11,16] of CTCs, in patients with early and metastatic BC.

CellSearch and other similar technologies, though, are limited to the detection of CTCs expressing the epithelial marker EpCAM. However, the invasion of tumor cells from peripheral blood is a complex process, during which tumor cells can undergo epithelial-to-mesenchymal transition (EMT) that involves the downregulation of epithelial markers, such as EpCAM, cytokeratins and E-cadherin, and the upregulation of mesenchymal markers, such as vimentin and N-cadherin. Several groups have shown the existence of non-epithelial CTCs and linked these subtypes to disease progression or other clinical-pathological features [17,18,19,20,21]. A plethora of new technologies emerged to overcome the limitations of EpCAM-based approaches, focusing on the challenge of detecting non-epithelial CTCs, mainly exploiting mesenchymal markers or physical features of cancer cells and other cancer-specific traits. Notably, few groups have specifically addressed altered metabolism of cancer cells [22,23,24,25,26,27]—recognized as a “hallmark of cancer” [28]—as a promising approach, also in view of the introduction of specific metabolic agent combined with chemo- or immunotherapies in several clinical trials [29,30,31]. One of the most described metabolic alterations of cancer cells consists of a markedly increased uptake of glucose in comparison with normal cells. First described by Otto Warburg in the 1920s, this cancer feature has been successfully exploited in the clinic by introducing a positron emission tomography (PET)-based imaging of the uptake of a radioactive glucose analog, which is commonly employed to stage cancer and assess response to therapy [32]. Several groups reported the exploitation of metabolic alterations to identify CTCs in the peripheral blood of metastatic cancer patients, independently from EpCAM expression and immunostaining techniques in general [22,23,24,25,26,27]. An mRNAseq study on CTCs from prostate cancer patients described a category of CTCs with an upregulation of metabolic transcripts as a potential biomarker for cancer metastasis [24]. Tang et al. [22] evaluated the presence of metabolically active CTCs in pleural effusion and in the peripheral blood of a limited number of non-small cell lung cancer patients by using the fluorescent glucose analogue 2-NBDG. This finding was also strengthened by our group in a preliminary study to assess the mutational status of hypermetabolic CTCs enriched by their elevated 2-NBDG uptake [23]. Other microfluidic platforms have been described for the screening of single-cell uptake of glucose or the release of by-product of metabolism such as lactate [33] or ROS [34].

In this study we focused on pH dysregulation [35,36,37]: in cancer, an augmented extracellular acidification is the consequence of both the increased lactate extrusion (in the context of the Warburg phenomenon) and of the sodium-proton antiporter NHE1 hyperactivity. pH dysregulation, an early phenomenon in carcinogenesis, is important for cancer cells to gain selective advantages and a more metastatic phenotype, and the acidic pH of the tumor milieu plays a recognized role in cancer invasion, metastasis, immunosuppression and therapeutic resistance [35]. Therefore, an abnormal metabolism constitutes an excellent candidate as a functional marker: since it represents an independent feature with respect to epithelial and mesenchymal markers, in principle, it could be leveraged for CTC detection without the need to identify specific phenotypic profile.

Droplet microfluidics can efficiently encapsulate single cells, maintaining cell growth and proliferation properties and enabling detection of secreted molecules, as they are retained inside the droplet [38,39]. We previously demonstrated that the different extracellular acidification rate (ECAR) of cancer cell lines with respect to normal white blood cells (WBCs) can be measured using a pH-sensitive dye inside microdroplets, allowing identification of cancer cells in peripheral blood [25]. As a proof of concept, we demonstrated the ability of the pH value to discriminate cancer cell lines from WBCs, and to detect CD45-negative cells with increased ECAR in patients with metastatic cancer of different types [25]. In this paper, we refer to this CTC detection method as “metabolism-based assay” (MBA). Here, we determined the prognostic role of MBA-CTCs in mBC and compared their performance with epithelial CTCs, as detected by the gold-standard CellSearch assay (CS-CTCs).

## 2. Results

### 2.1. Patients’ Characteristics

Between September 2016 and December 2017, 31 consecutive mBC patients were recruited at the Aviano National Cancer Institute, regardless the number and type of previous lines of treatment. CTCs were enumerated using both the metabolic-based assay (MBA) and CellSearch assay (CS) before a new line of treatment (T0) and after a median of three weeks following the first therapy cycle (T1). The CONSORT diagram of evaluable patients for each method is shown in Figure S1. Patient demographics and tumor characteristics at the time of enrollment are summarized in Table 1.

The patients’ median age was 56. Primary tumor receptor status for ER and/or PR (detected by IHC) and HER2 (evaluated by IHC or FISH) were positive in 21 (68%) and 3 (10%) out of 31 patients, respectively, while 8 (26%) out of 31 cases were triple-negative (ER-, PR- and HER2-negative).

Among the 31 patients recruited, 11 (35.5%) were starting their first line of therapy for metastatic disease; 28 (90.0%) received chemotherapy (alone or in combination with other treatments), 2 (6.5%) received hormone-therapy and 1 (3.2%) patient did not receive any treatment (control group of the A-BRAVE clinical trial, NCT02926196).

All patients had a minimal follow-up time of 12 months (median 18 months), and the first follow-up imaging study was performed on average 5.3 ± 2 months after the T0 blood sample collection.

First imaging re-evaluation after CTC enumeration documented a partial response/stable disease in 16 (59.3%) out of 27 patients with data; after the whole period of follow-up, instead, disease progression was observed in 25 (86.2%) out of 29 cases with data, and 13 (44.8%) patients deceased. Blood samples from 26 healthy donor (HD) volunteers were analyzed with MBA as negative controls.

### 2.2. MBA Description

**Figure 1:**
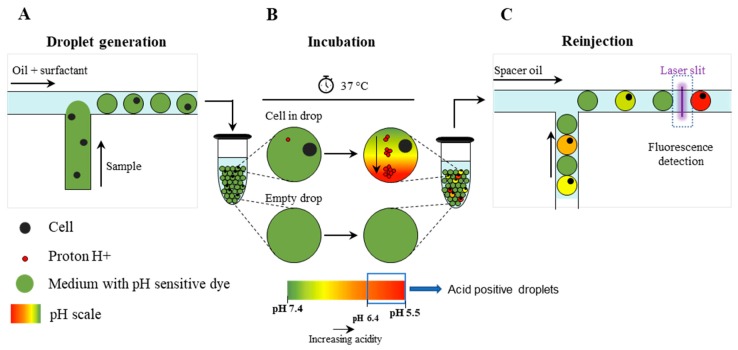
Schematic overview of the droplet microfluidic single-cell MBA for determination of the extracellular pH. (**A**) Single-cells are encapsulated into picoliter droplets together with an extracellular pH-sensitive dye (SNARF-5F); (**B**) During in-drop incubation, single cells secrete a certain quantity of protons (H+) which leads to a decrease of the pH value (i.e., acidification of the droplet content); (**C**) Droplets are reinjected into a second microfluidic device and screened for fluorescence by the optical setup.

The overall strategy adopted to detect cancer cells in a background of WBCs, by measuring the extracellular acidification rate (ECAR) [25], is outlined in Figure 1 and Figure S2. Briefly, single cells were encapsulated into 15 pL droplets together with a fluorescent pH-sensitive dye (SNARF-5F) (Figure 1A). During in-drop incubation, because of the cellular metabolic activity, each cell extruded a certain quantity of H+ altering the pH of the droplet (Figure 1B), which was then quantified by optical readout (Figure 1C and Figure S2). Beside pH, the system allowed to determine EpCAM and CD45 expression, as well as to record images of analyzed droplets to confirm the presence of a single cell within the droplet (Figure S2).

To determine a pH cut-off value for detecting breast cancer cells in mBC patients, both a basal-like, triple-negative (MDA-MB-231) and a luminal, hormone receptor positive (MCF-7) breast cancer cell lines, which are considered, respectively, representative of EpCAM-low and EpCAM-high [40] cell lines, were tested. By comparing breast cancer cells with WBCs, the cut-off value of pH < 6.4 granted detection of both breast cancer cell lines with a negligible number of WBC contaminants (0.045%, percentage which is further reduced in the patient analysis by CD45-labeling, Figure S3). Then, in subsequent analyses, cells that were negative for CD45 expression and within a droplet with pH < 6.4 were considered as MBA-CTCs.

In ex-vivo mBC blood samples, CTC count has been used to categorize patients as negative or positive for the presence of CTCs, if their count was 0 or ≥1, respectively. Moreover, a cut-off of 6 CTCs per 7.5 mL of blood was determined, adopting the same strategy that was used to set at 5 cells the prognostic cut-off value for the CS assay [10,15] (see Materials and Methods and Figure S4 for details); accordingly, patients were stratified as MBA-CTCs < 6 and MBA-CTCs ≥ 6.

### 2.3. Baseline CTC enumeration

Using the MBA assay, at T0, CTCs were detected in 12 out of 27 patients (44.4%), with 10 patients (37.0%) being above the cut-off value of 6 cells. Overall, the average CTC count was 218 ± 1022 (median 0, range 0–5319) (Table 2 and Table S1).

Among patients scored as CTC-positive, CTCs expressing EpCAM were detected in 6 (50.0%), with an average of 84 ± 90 (median 42, range 9–200), whereas EpCAM-negative CTCs were detected in 10 patients (83.3%), with an average of 539 ± 1612 (median 6, range 3-5125) (Table S1 and Figure S5).

In the cohort of HD volunteers, used as negative controls in this study, events with a metabolic signal comparable to CTCs could be detected in two (7.6%) out of 26, showing four and five cells, respectively (Table 2 and Table S2). Please note that these values are obtained by normalizing analysed sample volume to 7.5 mL for easier comparison with CS, as described in the materials and methods section. These two values correspond to one false positive event detected in each of these two HD in 1.9 and 1.5 mL of sample analyzed, respectively. Overall, HD cohort showed two false-positive events in more than 50 million analysed droplets. Notably, none of the HD showed more than six cells and the number of CTCs was significantly higher in mBC patients than in HDs (*p* = 0.001, Mann-Whitney U-test) (Figure S5).

By analyzing the same mBC population by CS, we found that 17 (77.3%) out of 22 evaluable patients had at least one CTC, while eight (36.0%) patients had a CTC number above the prognostic cut-off of five cells (as established in previous clinical studies) [10,15]. Overall, the average number of CTCs was 129 ± 433 (median 3, range 0–2022).

Moreover, we also determined the level of apoptotic CTCs by CS platform, as previously reported [41], by detecting in CTCs the expression of M30, an epitope of cytokeratin 18 revealed during early phase of apoptosis. Among the CTC-positive patients, three (17.6%) had at least one M30-positive CTC with an average count of 9 ± 14 (median 1, range 1–25) (Table S1).

### 2.4. Changes of CTC Levels After Starting Treatment

By the MBA, 15 (57.7%) out of 26 patients presented at least one CTC, while eight (30.7%) had more than six cells. With respect to T0, the overall average number of CTC decreased from 218 ± 1022 to 37 ± 75 (median 4, range 0–280) (Table 2 and Table S1). EpCAM-positive cells were found in seven (46.6%) MBA-CTC positive patients, with an average of 48 ± 65 (median 18, range 4–160) cells, whereas patients presenting EpCAM-negative CTCs were 12 (80%) out of 15, with an average of 52 ± 70 (median 14, range 3–225) cells (Table S1 and Figure S5).

Comparing CTC number at T0 and T1 for each patient, CTC level decreased in 10 cases and increased in six (Figure S6), while seven patients were negative at both time-points. The CTC concentration at T0 did not statistically differ respect to T1 (*p* = 0.2465, Wilcoxon test).

By CS analysis, at T1, 11 (50%) out of 22 evaluable patients had at least one detectable CTC, whereas five (23%) out 22 patients showed ≥5 CTCs. The average CTC number decreased from 129 ± 433 to 22 ± 65 (median 1, range 0–288) (Table 2 and Table S1).

Moreover, among the CTC-positive patients, apoptotic CTCs (M30-positive) were detected in 2 (18.2%) out of 11 sample, accounting for one and seven cells, respectively. Overall, the number of M30-positive cells at both time-points was so low that it did not allow reliable data analysis.

Among the 17 patients that had CS paired samples at T0 and T1, 2 (11.8%) had an increase and 10 (58.8%) a decrease in CTC levels, while 5 (29.4%) cases showed unchanged CTC value (Figure S6). Unlike MBA results, CS-CTC levels significantly differed between T0 and T1 (*p* = 0.0146, Wilcoxon test).

### 2.5. Comparison of CTC Levels Using MBA and CS

**Figure 2:**
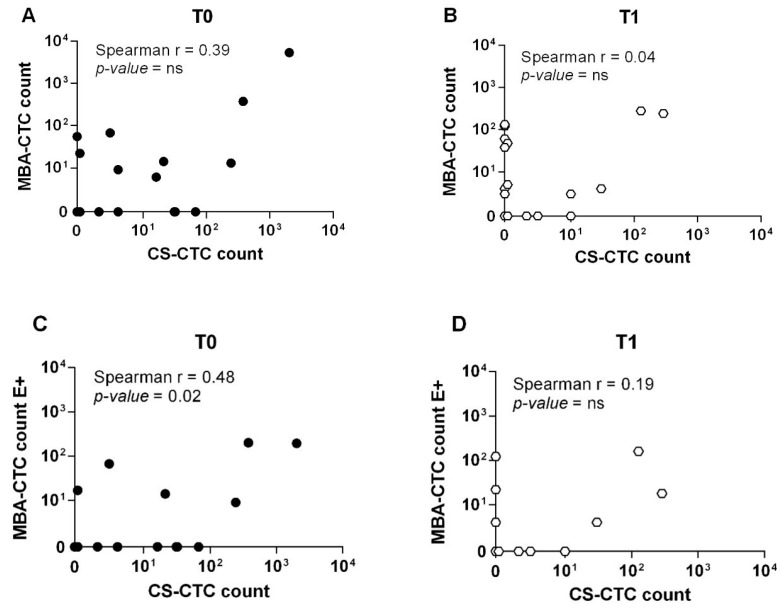
Direct comparison of CTC enumeration between the MBA and the CellSearch method. (**A**) Scatterplot of CTC count as detected by the MBA and the CS method at baseline (T0) and (**B**) follow-up (T1). (**C**) Scatterplot of EpCAM-positive CTC as detected by the MBA and the CS methods at baseline (T0) and (**D**) follow-up (T1). Only complete pairs of data including results from both MBA and the CS method were plotted. Overall, 22 and 21 cases had matched blood sample at T0 and T1, respectively.

A total of 22 patients were analyzed in parallel with both the MBA and the CS at T0 and 21 patients at T1. The total number of patients with a CTC level < or ≥ the cut-off for each technique at each time-point is reported in Table 3. Using the respective prognostic cut-offs (MBA: ≥ 6 CTCs; CS: ≥ 5 CTCs) and considering both EpCAM-positive and EpCAM-negative cells detected by MBA and CS-CTCs, the overall positive concordance was 68.2% at T0 and 61.9% at T1 (Table 3, left panel) and no significant correlation between matched samples was found (T0: Spearman *r* = 0.39, *p-*value = ns; T1: Spearman *r* = 0.04, *p-*value = ns) (Figure 2A,B); considering only EpCAM-positive MBA (MBA-E+) CTCs and CS-CTCs, instead, concordance analysis revealed a positive conordance of 72.7% and 76.2% at T0 and T1 (Table 3, right panel), respectively, while there was a significant correlation with CS at T0 (Spearman *r* = 0.48, *p-*value = 0.02) but not at T1 (Spearman *r* = 0.19, *p-*value = ns) (Figure 2C,D).

Additionally, a paired test comparing the CTC numbers detected by CS and MBA was performed (nonparametric, Wilcoxon matched-pairs signed rank test). The Pratt modification to the original Wilcoxon test was used, in order to take into account the large number of cases in which both MBA and CS scored equally, detecting 0 cells. *p-*value was not significant both at T0 (*p-*value = 0.07) and T1 (*p-*value = 0.39), indicating that the median of the two groups (MBA vs. CS) did not significantly differ.

### 2.6. Concordance Between CTC Status and Radiologic Imaging

**Figure 3:**
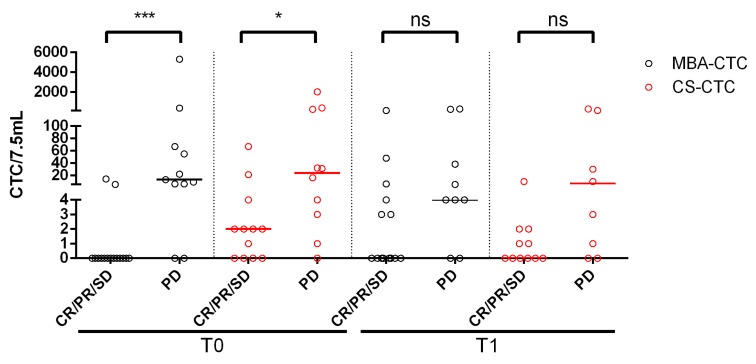
Comparison between CTC levels as detected by the metabolic-based assay (MBA) and CellSearch (CS) and imaging. This plot shows the CTC count for individual patients at baseline (T0) and follow-up (T1), compared with their response at the first follow-up imaging. (CR/PR/SD = Complete Response/Partial Response/Stable Disease; PD = Progressive Disease. Statistical significance was calculated by Mann-Whitney test (*** *p* = 0.0001, * *p* = 0.027, ns = not significant (CS-CTCs: *p* = 0.05; MBA-CTCs: *p* = 0.07).

The agreement between the number of CTCs assessed at T0 and T1 and the presence of complete response/partial respose/stable disease (CR/SD/PR) or progressive disease (PD) at the first follow-up imaging study was tested. For MBA-CTCs, a good agreement (kappa = 0.761) at T0 and a poor agreement (kappa = 0.123) at T1 were observed. CS showed a moderate agreement both at T0 (kappa = 0.441) and at T1 (kappa = 0.431). In the best case (MBA-CTC at T0), 88.5% of therapy responses was correctly predicted by MBA. Results are shown in Figure 3 and Table S3.

### 2.7. Survival Analysis

**Figure 4:**
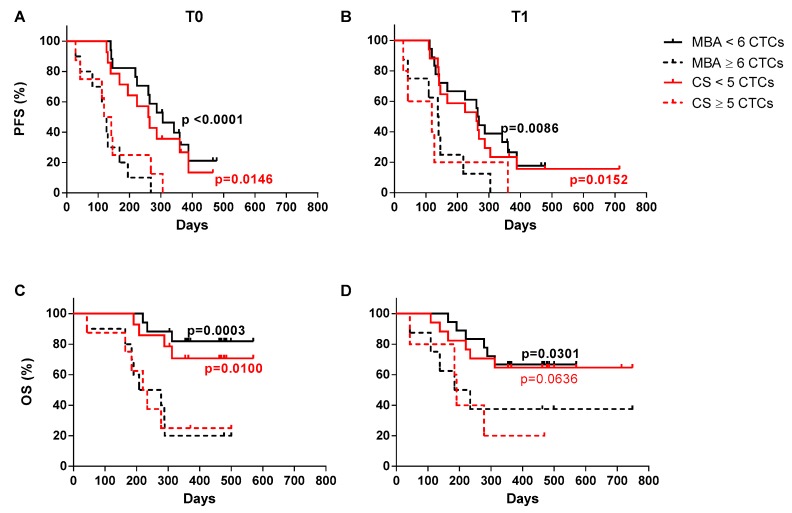
Progression-free survival (PFS) and overall survival (OS) of mBC patients. (**A**) Kaplan-Meier plots estimating PFS at baseline (T0) and (**B**) first follow-up (T1) by the metabolic-based assay (MBA) and the CellSearch (CS) method. (**C**) Kaplan-Meier plots estimating OS at baseline (T0) and (**D**) first follow-up (T1) by the MBA and the CS method. For these analyses, mBC patients were stratified using a cut-off value of 6 CTCs for the MBA and 5 CTCs for the CS.

As shown in Figure 4, progression-free (PFS) and overall survival (OS) were predicted at T0 and T1 for both methods, when patients were stratified according to the respective prognostic cut-off values. Patients analyzed by MBA and stratified according to CTC number ≥6 or <6, showed a significantly different median PFS at both T0 and T1 (Figure 4A,B). Similarly, MBA-CTC level ≥6 or <6 was able to predict OS at both T0 and T1 (Figure 4C,D). Conversely, the CS results were only partially overlapping with MBA data. Indeed, the CS-CTC number ≥5 or <5 predicted PFS (Figure 4A,B) at both the examined time-points, and OS at T0, but not at T1 (Figure 4C,D, and Table 4). Notably, an exceptionally high number of CTCs characterized two patients with an extremely short survival, at both time-points with both techniques.

#### 2.7.1. Patient Stratification According to the Combined Application of the Two CTC Detection Technologies

Since the MBA and the CS test are likely based on different biological peculiarities of cancer cells, the hypothesis that the two techniques might unveil additional information enabling a more accurate stratification of patients, when combined, was tested.

**Figure 5:**
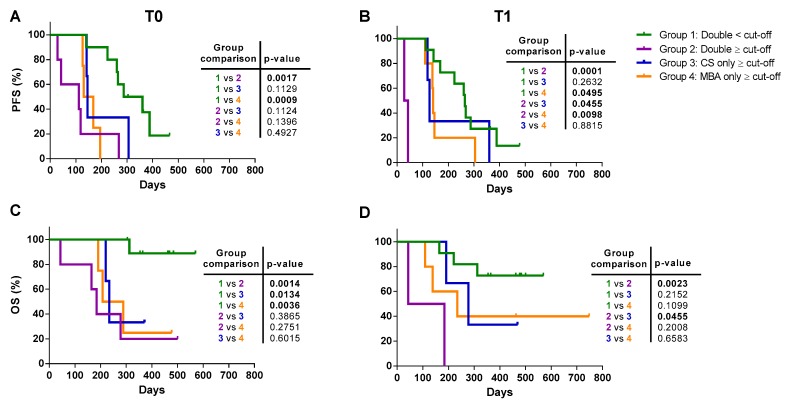
Progression-free survival (PFS) and overall survival (OS) of mBC patients stratified by the combination of the metabolic-based assay (MBA) and CellSearch (CS) method. The patients were stratified into four group: group 1 (green curve), patients with a CTC count below the cut-off with both methods; group 2 (violet curve), patients with a CTC count ≥ cut-off with both methods; group 3 (blue curve), patients with a CTC count ≥ cut-off only for the CS; and group 4 (yellow curve), patients with CTC count ≥ cut-off only for the MBA. (**A**) PFS of mBC patients stratified by the combination of the two methods at baseline (T0) and (**B**) follow-up (T1). (**C**) OS of mBC patients stratified by the combination of the two methods at baseline (T0) and (**D**) follow-up (T1). Statistical analysis of PFS and OS between the four groups are reported in the embedded tables.

Thus, according to the cut-off of six and five CTCs for the MBA and CS, respectively, patients were stratified into four groups: 1) Patients who had in both tests a CTC count lower than the respective reference cut-off (double < cut-off) or 2) equal or higher than it (double ≥ cut-off); 3) patient with CS-CTC ≥ cut-off, and MBA-CTC < cut-off (CS only ≥ cut-off) and 4) patients with MBA-CTCs only ≥ cut-off and CS-CTC level < cut-off (MBA only ≥ cut-off). Kaplan-Meier curves for PFS and OS are shown in Figure 5 and median PFS and OS are summarized in Table 5.

Overall, patients with a CTC count double < cut-off had the best prognosis, with a higher median PFS (Figure 5A,B) and OS with respect to all the other groups (Figure 5C,D). Patients classified as double ≥ cut-off showed similar PFS to CS only ≥ cut-off and MBA only ≥ cut-off patients at T0 (all these curves were not significantly different among them, but only vs. double < cut-off) (Figure 5A). Moreover, at T1, double ≥ cut-off patients had the worst PFS, significantly different vs. all other curves (all differences *p* < 0.05) (Figure 5B).

With respect to OS, double ≥ cut-off and patients with a CTC count ≥ cut-off with only one technique had similar median OS at T0 (Figure 5C), but, at T1, none of double ≥ cut-off patients were still alive after follow-up, differently from all other cases (Figure 5D). These data suggest that the clinical information provided by the two detection methods are different and, by combining the results obtained with the two technologies, a more accurate patient stratification could be achieved.

It is worth noticing that, despite the two tests had a similar percentage of patients above the cut-off value, the two CTC populations were only partially overlapping: there were patients who presented a number of CTC ≥ cut-off with one test but not the other one (T0: 41% for MBA vs. 36% for CS; T1: 33% for MBA vs. 24% for CS), whereas the prevalence of patients with a CTC count ≥ cut-off in at least one test was 54.5% at T0 and 47.6% at T1. Thus, overall, the combined results of the two tests showed a ~50% increase in the prevalence of patients with CTCs ≥ cut-off with respect to that observed with each single test alone. Survival curves for these two populations (double ≥ cut-off or at least one ≥ cut-off) were almost similar but with the benefit of detecting a higher number of patients with a number of CTCs above the defined cut-off.

#### 2.7.2. Survival Analysis According to the CTC Status Before and After Therapy

**Figure 6:**
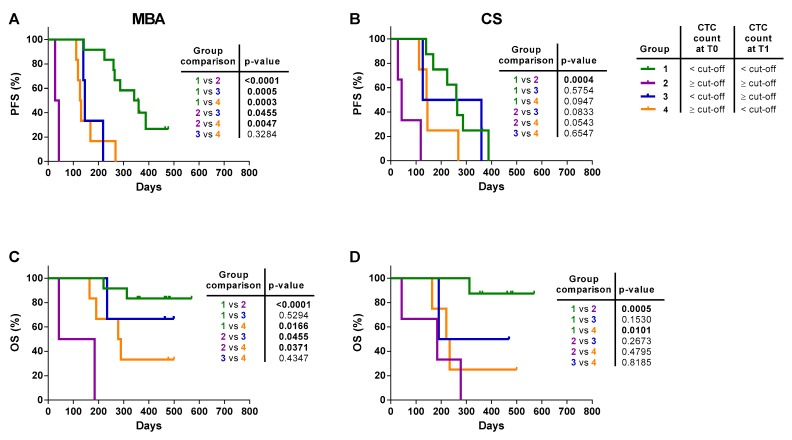
Progression-free survival (PFS) and overall survival (OS) of mBC patients stratified by the level of CTC before and after therapy. Patients were stratified into four groups: Group 1 (green curve), patients with a CTC count < cut-off both at T0 and T1; Group 2 (violet curve), patients with a CTC count ≥ cut-off both at T0 and T1; Group 3 (blue curve), patients who presented a CTC count below and equal and higher than the cut-off at T0 and at T1, respectively; and group 4 (yellow curve), patients with a change of CTC count from above to below the cut-off at T0 and T1, respectively. (**A**) Kaplan-Meier plots of changes in CTC level from baseline (T0) and the first follow-up (T1) to predict PFS with the metabolic-based assay (MBA) and (**B**) the CellSearch (CS). (**C**) Kaplan-Meier plots of changes in CTC level from baseline (T0) and the first follow-up (T1) to predict OS with the MBA and (**D**) the CS. Statistical analysis of PFS and OS between the 4 groups are reported in the embedded tables.

To investigate whether a change in the level of CTCs could be used as a predictor of rapid progression and death, patients were stratified according to the CTC status before and after the start of therapy, according to the cut-off of six or five CTCs for the MBA and CS, respectively. Thus, four different groups of patients were compared: Group 1, patients having a persistently CTC count < cut-off or, Group 2, persistently ≥ cut-off; Group 3, patients with a CTC count < cut-off at T0 who converted to a CTC level ≥ cut-off post-therapy (T1); and Group 4, patients with a CTC count ≥ cut-off at T0 but who had a conversion to a CTC number < cut-off at T1 (Figure 6).

Patients with the MBA-CTC count ≥ cut-off at both time-points had the worst prognosis compared to patients with a CTC count < cut-off at both time-points. Patients with CTC count ≥ cut-off either at baseline or at follow-up had an intermediate and statistically different outcome compared to Group 1 and Group 2 (Figure 6A). In CS analysis, similarly, patients with ≥ 5 CTCs at both time-points had the shortest median PFS, significantly different to patients with a persistently < 5 CTC level. Group 3 and 4 did not reach statistical difference with respect to both Group 1 and Group 2 (Figure 6B and Table 6).

As shown in Figure 6C,D, regardless of the CTC detection method, the risk of death for those patients who never had a CTC count ≥ cut-off was lower, with ~80% of patients still alive at the end of the observational periods, compared to patients with a CTC count ≥ cut-off at both time points, all dead at the end of the observational time. Interestingly, patients with only one MBA-CTC count ≥ 6, with respect to patients with a CTC count persistently ≥ 6 or < 6, presented an intermediate prognosis. This difference was not evident in patients with only one CS count ≥ 5 CTCs, being the prognosis of these patients not significantly different from that with ≥ 5 CTCs at both time points.

## 3. Discussion

Studies of other groups involving CTC detected with a metabolism-based approach did not correlate results with clinical outcome. In the present pilot study, we determined that elevated MBA-CTCs are linked to shorter overall and progression-free survival. Additional information was obtained considering CTC levels over time.

One of the limitations of this study is the relatively small cohort involved, though our cohort reflects general population in terms of cancer subtype prevalence (luminal, HER2-positive, triple-negative), metastatic sites and lines of therapy. This is further confirmed by the results obtained in this study with CS, which are aligned with larger clinical trials in metastatic breast, colon and prostate cancers [10,11,15,16,42,43]. Despite the relatively small cohort, results display unequivocal statistical significance. Thus, we feel confident enough stating that the presented results offer a first evidence that MBA-CTCs are prognostically informative across different subtypes of mBC patients. Further validation in independent studies is mandatory, because survival analysis was conducted in the same population on which cut-off was assessed.

Another limitation is the relatively small volume of sample analyzed (2.5 mL vs. the usual 7.5 mL). We calculated and analyzed cumulative Poisson distribution for known CTC concentrations ranging from 2 to 30 cells/7.5 mL to evaluate how a smaller sampling volume could affect accuracy of CTC estimation. The analysis is descripted in supplementary materials (Figure S7). Overall, a smaller sampling volume affects accuracy of CTC detection to a slight extent (1–2 cells of difference for CTC values around 2/7.5mL, 5–6 cells of difference for CTC values around 30/7.5mL). However, this in most cases does not interfere with deeming the patient positive or negative with respect to cut-off. For values close to cut-off, variability of patient classification is similar between the two sampling volumes, with an increased uncertainty for a narrow range of concentrations (close to 10 cells/7.5 mL).

Interestingly, the agreement between cells detected by MBA and CS was relatively weak and no significant correlation was found, while both methods independently yielded similar prognostic significance. Moderate correlation and fair agreement were found instead for EpCAM-positive cells detected by the two methods suggesting partial overlapping of EpCAM population. In other words, it seems like some, but not all, of EpCAM-positive CK-positive cells display anormal metabolism, while some, but not all, of EpCAM-positive MBA-positive cells display cytokeratins. By combining data obtained by MBA and CS, additional information could be gained, suggesting that the two techniques detect different CTC subgroups with complementary information, possibly reflecting different aspects of disease evolution. This interpretation is strengthened by the fact that the prevalence of single-positive patients (either MBA or CS above cut-off) is roughly 50% higher than the prevalence obtained considering single MBA or CS testing. A possible explanation is the fact that metabolism is altered also in non-epithelial cells [44], which are mostly EpCAM-negative and thus not detected by CS. At the same time, our pH threshold was relatively stringent in order to maximize purity, since it was set in order to obtain absence of dysmetabolic cells in healthy controls or absence of WBCs contaminants in other words. This could lead to “missing” of some EpCAM-positive cells with increased ECAR with respect to normal WBCs, but not enough increased to meet pH threshold.

It should be now clear that referring to CTCs without further definition might be confusing, as it is a rather blurred group of cells of different nature and maybe distinct clinical implications. More studies are needed to clearly understand if different types of cells could be linked to specific clinical and pathological features or therapy susceptibilities.

Hypothesizing a predictive role of MBA-CTCs, a growing body of evidence suggests that the acidic extracellular environment of tumor tissues confers chemoresistance: indeed, a majority of chemotherapeutics (e.g., doxorubicin) behaves like weak bases which are quickly protonated in low pH environments, leading to a reduced cellular uptake and treatment efficacy [45]. Thus, restoring a physiologic extracellular pH is emerging as a new therapeutic strategy to increase tumor chemosensitivity to traditional chemotherapeutics. Several pH regulator inhibitors have already been evaluated in several clinical trials [30,46] and, in this contest, MBA-CTCs might represent a fitting approach to monitor the benefit of this new therapeutic strategy.

CTCs identified by their aberrant metabolism seem at least as tightly linked to survival and disease progression as epithelial CTCs, either when measured before or after the therapy, and even more when considered over time; it emerged that MBA-CTCs provide different information with respect to CS-CTC, which can be combined to increase accuracy in mBC prognostic stratification. Overall, this pilot study can be used as a foundation to design larger clinical studies needed to confirm and validate MBA-CTCs as prognostic/predictive biomarkers.

## 4. Materials and Methods

### 4.1. Study Design

This pilot study trial was conducted comparing the metabolism-based assay (MBA) with the CellSearch (CS) system to test prognostic meaning of CTC enumeration. The study was conducted at the IRCCS-CRO Aviano-National Cancer Institute and approved by the Institutional Review Board with number IRB-12-2014. Informed and written consent was obtained from all patients and healthy donors before their enrolment, and their clinico-pathological information was recorded. Thirty-one patients with progressive and measurable mBC, at the beginning of a new systemic therapy, without limits to round and type of previous therapies (hormone therapy, chemotherapy, targeted therapy) were included. All patients had an Eastern Cooperative Oncology Group performance status (ECOG PS) score ≤ 1.

Before starting a new therapy, patients underwent a baseline (T0) blood drawn for CTC evaluation and routine clinical tests. Another blood sample was collected 3–4 weeks after the beginning of the therapy (follow-up, T1). Among the 31 enrolled patients, 27 and 26 blood samples were evaluated for the presence of CTC with the MBA at T0 and T1, respectively, whereas CTC count was evaluable with the gold standard CS for 22 patients at both time points. Details about reasons for exclusion (e.g., insufficient or clotted blood sample, organizational and/or technical failure) are summarized in Figure S1.

Clinical re-evaluation of the disease status was conducted depending on the type and schedule of the therapy; Standard Response Evaluation Criteria in Solid Tumors (RECIST) criteria were used to determine patients’ responses to treatment. This study did not interfere with routine imaging schedule, nor imposed a uniform imaging schedule to all patients. The follow-up imaging schedule was decided by the oncologists on an individual basis. For most patients entered in the study, CT of the chest and abdomen, or PET, approximately every 3–6 months, were performed. In selected cases e.g., in the case of liver metastases as the unique site of disease, the disease parameter was simplified by using a liver ultrasound.

### 4.2. Microfluidic Platform

#### 4.2.1. Device Fabrication

In this work, a microfluidic device was employed for droplet generation, and a second one for droplet reinjection in order to screen their fluorescence for pH determination and antibody staining. Each device was made of PDMS bonded to a glass surface, as previously reported [27].

#### 4.2.2. Optical Setup

The optical setup for measuring droplet fluorescence consisted of an IX70 inverted fluorescence microscope (Olympus, Tokyo, Japan). A 25 mW, 405 nm laser beam was focused with a cylindrical lens crossing orthogonally the microfluidic channel. Fluorescence signal emitted from droplets was captured by a 40× objective (Olympus LUCPlanFLN, 40×/0.60), split with dichroic filter and detected through bandpass filters (579/34; 630/38 e 494/20, 435/20) by photomultiplier tubes (PMTs) (H957–15, Hamamatsu, Hamamatsu city, Japan). Signal went through a transimpedance amplifier with 1V/uA gain and detected by the acquisition system (cRIO-9024, National Instruments, Austin, TX, USA, analog input module NI9223) with a 10 μsec scan rate.

#### 4.2.3. Droplet Generation and Encapsulation of Cells

The emulsification device was used to generate droplets. This device has a flow-focusing junction, where the aqueous phase (cell suspension) and carrier oil stream meet, and droplet generation occurs (forming a water-in-oil emulsion). The resulting emulsion flowed off-chip through PTFE tubing (Adtech Polymer Engineering, Stroud, UK) connected to the outlet of the device and collected into 1.5 mL vial (Eppendorf, Hamburg, Germany) placed on an ice-cold rack (IsoTherm System, Eppendorf). The vial containing droplets was incubated for the desired incubation time at 37 °C. After incubation, droplets were immediately cooled by replacing the vial on the ice-cold rack and reinjected into the required microfluidic device for subsequent analysis.

#### 4.2.4. Droplet Fluorescence Screening, Data Acquisition and Control System

Droplets were reinjected into the emulsion device through PTFE tubing (Adtech Polymer Engineering, Stroud, UK). The data acquisition system has a 10 μsec scan rate. The signal output voltage of each droplet was recorded and processed in real time by a field programmable gate array (FPGA) card. Droplets with a size within defined range and gated fluorescence were imaged by synchronous triggering of an LED and a CMOS camera, and (optionally) sorted with a dielectrophoretic pulse. Data are transferred from the FPGA to the real-time controller providing a record of images and raw tracks for each run. A custom LabVIEW software allows the operator to access the picture gallery, evaluate the pictures of droplets to discard droplets that are empty or containing particles not compatible with the morphology of intact cells (debris).

### 4.3. pH-Assay for Extracellular Acidification Measurements

The pH-sensitive fluorescent dye SNARF-5F (Invitrogen-Thermo Fisher Scientific, Waltham, MA, USA) was used to measure the pH of each droplet. SNARF-5F responds to pH variations by undergoing a wavelength shift in the emission spectra. Such pH-dependent shifts allow the ratio of the fluorescence intensities from the dye at two emission wavelengths (580 nm and 630 nm) to be used for quantitative determination of pH. For each droplet the ratio of emitted fluorescence intensities at 580 and 630 nm (580/630 ratio) of SNARF-5F is calculated in real time. As the pH is more acidic, SNARF-5F fluorescence increases at 580 nm while decreases at 630 nm. To calibrate the system, Joklik’s EMEM medium titrated at different pH (7.4, 7, 6.5, 6, 5.5 and 5) was added with 4 µM SNARF-5F, emulsified and droplets screened for fluorescence. The 580/630 nm ratio were calculated for each pH and plotted against the respective pH, which allowed constructing a calibration curve.

### 4.4. Cell Lines

Breast cancer cell lines MDA-MB-231 and MCF7 were obtained from the American Type Culture Collection (Manassas, VA, USA) and cultured in DMEM medium (Sigma, St. Louis, MO, USA) supplemented with 10% FBS, in a humidified atmosphere at 37 °C and 5% CO_2_. Cells used were detached before confluency, tested for absence of mycoplasma contamination and validated for short tandem repeat profiling.

### 4.5. Identification of a ph Threshold to Discriminate CTC from WBC in Patient Samples

To identify a threshold of extracellular acidification rate (i.e., pH) able to better discriminate between CTCs and WBCs, the ECAR of breast cancer cell lines (MDA-MB-231 and MCF7), and WBCs was separately assessed by using the MBA. WBCs were obtained from the peripheral blood of healthy donors (n = 3), after red blood cell lysis with BD Pharm Lyse lysing solution (Beckton-Dickinson, Franklin Lakes, NJ, USA) according to the manufacturer’s instructions, while breast cancer cell line (MCF7 and MDA-MB-231), grown as previously described at a confluence of 70–80%, were detached with 1% Trypsin-EDTA. Cells were resuspended at a concentration of 1,000,000 cells/mL in 50 μL of the unbuffered Joklik’s modified EMEM culture medium (Sigma) containing 2 mM EDTA, 0.1% BSA, 15% Optiprep and 4 mM of the fluorescent pH indicator SNARF-5F, emulsified as described in the previous section, incubated at 37 °C for 30 min and analyzed. All experiments were performed in triplicate at various cell passages.

### 4.6. CTC Detection by the MBA

Blood samples were drawn into K2-EDTA Vacutainer tubes (Beckton Dickinson) and maintained at room temperature. For each sample, 2.5 mL of blood were analyzed within 2 h after collection. Red blood cells (RBC) were lysed with the BD Pharm Lyse lysing solution (Beckton-Dickinson, Franklin Lakes, NJ, USA), according to the manufacturer’s instructions, and centrifuged at 200 RCF for 5 min. Thereafter, the nucleated fraction was depleted of CD45-positive WBCs and residual RBCs using CD45 and Glycophorin A microbeads (Miltenyi Biotec, Bergisch GladBack, Germany), respectively, and LD separation columns in a MACS MIDI separator (Miltenyi Biotec), according to the manufacturer’s instructions. The CD45-negative cells were recovered, centrifuged at 300 RCF for 10 min and stained with anti-CD45 (BD Horizon Brilliant™ Violet 480, dilution 1:100) and anti-EpCAM (BD Horizon Brilliant™ Violet 421, dilution 1:100) for 25 min at room temperature. After washing the sample with PBS-BSA 0.5%, cells were resuspended in 50 μL of the unbuffered Joklik’s modified EMEM culture medium (Sigma) containing 2 mM EDTA, 0.1% BSA, 15% Optiprep and 4mM of the fluorescent pH indicator SNARF-5F (Thermo Fisher Scientific). Then, cells were single-cell encapsulated in monodispersed droplets using the droplet microfluidic platform as described in previous section. The emulsion was collected and incubated at 37 °C and 5% CO_2_ for 30 min and then reinjected into the microfluidic channel for fluorescent reading of pH, CD45 and EpCAM expression, and the acquisition of bright-field imaging of each positive event. Positive events were defined as CD45-negative cells able to acidify their extracellular environment to a pH lower than 6.4 (MBA-CTC). The number of CTCs was then proportionally adjusted to 7.5 mL for an easier comparison to the one reported by the CS test. This was done by obtaining the total volume of sample analyzed (Technical note: the starting volume of blood analyzed was always the same: 2.5 mL. However, in the microfluidic analysis, a variable fraction of volume is usually lost for technical reasons (tuning of parameters at the beginning of the acquisition, junk accumulating at the beginning/end of the sample) or adverse events (clogging of microfluidics and restart of data acquisition). Fortunately, droplet microfluidics technology allows the operator to know the precise volume analyzed, because the number of total droplets analyzed and the volume of droplet is known. Therefore, the operator can always calculate the proportion of detected events with respect to the analyzed volume) (total volume = droplet volume × number of droplets analyzed) and doing the proper proportion (MBA-CTC (measured)/volume analyzed = MBA-CTC/7.5 mL). All evaluations were performed without knowledge of the clinical status of the patients.

### 4.7. CTC Detection by CS

Blood samples were drawn into 10 mL CellSave Preservative Tubes (Menarini) and maintained at room temperature. Blood samples were sent to Veneto Institute of Oncology IOV-IRCCS, processed and analyzed according to manufacturer’s instruction [13]. To quantify the fraction of apoptotic CTCs, a FITC-conjugated anti-M30 monoclonal antibody was used in conjunction with the standard CTC kit, for recognizing a neoepitope in cytokeratin 18 (CK18) that becomes available at a caspase cleavage event during apoptosis, and is undetectable in viable epithelial cells as previously described [41]. All evaluations were performed without knowledge of the clinical status of the patients.

### 4.8. Statistical Analysis

Data were analyzed using GraphPad Prism 6 (version 2.6, San Diego, CA, USA). Patient and clinical characteristics were presented as frequency and percentage, median and range, mean and standard deviation (SD), as appropriate. Comparison of median between groups were performed by Mann-Whitney test and groups were compared using the Wilcoxon rank test, as appropriate. PFS and OS (time elapsed from enrolment to disease progression and death from any cause, respectively) were determined by Kaplan–Meier plots, with data being censored at last follow-up if progression or death had not occurred. Gehan-Breslow-Wilcoxon test were used to compare the survival curves by CTC detection groups. *p* < 0.05 was considered significant.

### 4.9. Cut-Off Determination for ECAR Method

Median PFS times and Cox proportional hazard ratios over all possible CTC cut-offs using baseline data were used to determine the optimal CTC cut-off for the prediction of PFS. The median PFS for the patients above or below each cut-off was calculated. In addition, the percentage of patients above each CTC cut-off was calculated. The best cut-off was selected as the one with the highest Cox hazard ratio, being above the normal background and having at least 40% patients above the cut-off. A CTC count of six or more per 7.5 mL of blood is predictive of shorter PFS. The chart showing median PFS for each cut-off is shown in Figure S4. The cut-off of six CTCs/7.5 mL was determined using only results from the baseline and PFS as the outcome. Additional analyses have shown that the cut-off for other variable and time points (OS at the baseline, PFS and OS at 1st follow-up) may differ. In order to show uniform results and simplify the interpretation, a CTC cut-off of six CTC/7.5 mL was used for all analyses.

## 5. Conclusions

In this pilot study, cellular pH dysregulation is exploited in an attempt to detect CTCs in metastatic breast cancer. Results suggest that metabolically aberrant CTCs, detected with the method presented herein, provide useful prognostic information. Clinical validity needs further confirmation in larger cohorts of patients.

## 6. Patents

Patent number ITRM20130700A1, 19 Dec 2013. Patent family ID 50073355 (Published as CN105849559A; CN105849559B; EP3084434A1; EP3084434B1; ES2673597T3; WO2015092726A1; ITRM20130700A1; JP2017502312A; JP6437009B2; US2017003306A1; US9958463B2).

## Figures and Tables

**Table 1:** Patient and tumor characteristics.

Characteristics	N	%
All patients	31	100.0
Progressed	25	80.6
Alive	18	58.0
Age at baseline (years)		
Median	56	
Range	39–78	
ER- and PR-receptor status		
ER+ or PR+	21	68.0
ER− and PR−	10	32.0
Her2/neu status		
Positive	3	10.0
Negative	28	90.0
Triple negative	8	26.0
Sites of metastasis		
Visceral	21	68.0
Non-visceral	10	32.0
Bone	23	74.0
Lung	14	45.0
Brain	2	6.0
Liver	14	45.0
Nodes	19	61.0
Number of metastasis		
1	9	29.0
2 or more	22	71.0
Therapy		
First line	11	35.5
Second line or subsequent	20	65
Type of therapy		
Chemotherapy alone	20	64.5
Chemotherapy and targeted therapy	8	25.8
Hormone-therapy	2	6.5
Placebo	1	3.2

**Table 2:** Prevalence of CTCs at baseline (T0) and follow-up (T1) as detected by the metabolism-based assay (MBA) and CellSearch (CS), according to the cut-off of 6 and 5 CTCs, respectively. (HD = healthy donor volunteers; mBC = metastatic breast cancer patients).

Cohort	N	Mean ± SD	Median	Range	% of Patients with CTCs Above Cut-Off
MBA					
HD	26	0 ± 1	0	0–5	0%
mBC at T0	27	218 ± 1022	0	0–5319	37%
mBC at T1	26	37 ± 75	4	0–280	30.7%
CS					
mBC at T0	22	129 ± 433	3	0–2022	36.4%
mBC at T1	22	22 ± 65	1	0–288	22.7%

**Table 3:** Concordance of CTC status between the metabolism-based assay (MBA) and CellSearch (CS) method at baseline (T0) and follow-up (T1), using respective cut-offs.

Left Panel						Right Panel					
Time-Point	Cell Population	Cut-Off	CS		Tot	Time-Point	Cell Population	Cut-Off	CS		Tot
			<5	≥5					<5	≥5	
*T0* ^a^						*T0* ^c^					
	MBA	<6	10	3	13		MBA-E+	<6	12	4	16
		≥6	4	5	9			≥6	2	4	6
		Tot	14	8	22			Tot	14	8	22
*T1* ^b^						*T1* ^d^					
	MBA	<6	11	3	14		MBA-E+	<6	14	3	17
		≥6	5	2	7			≥6	2	2	4
		Tot	16	5	21			Tot	16	5	21

^a^ Kappa = 0.330 (fair agreement); SE of kappa = 0.205; 95% CI from −0.071 to 0.732; Number of observed agreements: 15 (68.2% of the observations); ^b^ Kappa = 0.077 (poor agreement); SE of kappa = 0.218; 95% CI from −0.351 to 0.504; Number of observed agreements: 13 (61.9% of the observations); ^c^ Kappa = 0.377 (fair agreement); SE of kappa = 0.205; 95% CI from −0.025 to 0.780; Number of observed agreements: 16 (72.7% of the observations); ^d^ Kappa = 0.295 (fair agreement); SE of kappa = 0.243; 95% CI from −0.180 to 0.771; Number of observed agreements: 16 (76.2% of the observations).

**Table 4:** PFS and OS by CTC count for metabolism-based assay (MBA) and CellSearch (CS) method at baseline (T0) and follow-up (T1), using respective cut-offs.

Group	MBA-CTC < 6	MBA-CTC ≥ 6	*p*-Value	CS-CTC < 5	CS-CTC ≥ 5	*p*-Value
*T0*						
N (%)	17 (63)	10 (37)		14 (63.6)	8 (36.4)	
Median PFS (days)	306	123	<0.0001	262	131	0.0146
Median OS (days)	>600	243	0.0003	>600	227	0.01
*T1*						
N (%)	18 (69.2)	8 (30.8)		17 (77.3)	5 (22.7)	
Median PFS (days)	266	139	0.0086	260	119	0.0152
Median OS (days)	>600	209	0.0301	>600	191	0.0636

**Table 5:** PFS and OS according to the combined application of the metabolism-based assay (MBA) and CellSearch (CS).

Group	1-Double < Cut-Off	2-Double ≥ Cut-Off	3-CS Only ≥ Cut-Off	4-MBA Only ≥ Cut-Off
*T0*				
N (%)	10 (45.5)	5 (22.7)	3 (13.6)	4 (18.2)
Median PFS (days)	323.5	112	146	149.5
Median OS (days)	> 600	184	234	248
*T1*				
N (%)	11 (52.4)	2 (9.5)	3 (14.3)	5 (23.8)
Median PFS (days)	264	35.5	127	140
Median OS (days)	>600	113.5	278	234

**Table 6:** PFS and OS by the CTC status before and after therapy for the metabolism-based assay (MBA) and the CellSearch (CS).

Group	1-CTC < Cut-Off at All Time Points	2-CTC ≥ Cut-Off at All Time Points	3-CTC < Cut-Off at T0 + CTC ≥ Cut-Off at T1	4-CTC ≥ Cut-Off at T0 And CTC < Cut-Off at T1
MBA				
N (%)	12 (52.2)	2 (8.7)	3 (13.0)	6 (26.1)
Median PFS (days)	351	35.5	146	129
Median OS (days)	>600	113.5	>600	283
*CS*				
N (%)	8 (47.1)	3 (17.6)	2 (11.8)	4 (23.5)
Median PFS (days)	262	43	243.5	144
Median OS (days)	>600	184	469.5	227

## Supplementary Materials

The following are available online at https://www.mdpi.com/2072-6694/12/4/1005/s1. Figure S1. CONSORT diagram describing the study design, enrolment of patients and motivations of data exclusion, Figure S2. Principle of the metabolic-based assay (MBA) CTC detection, Figure S3. Measurement of ECAR by the metabolism-based assay (MBA), Figure S4. Determination of the prognostic CTC cut-off for the metabolic-based assay (MBA), Figure S5. CTC count of healthy donors (HD) and mBC patients at baseline (T0) and follow-up (T1) performed with the metabolic-based assay (MBA), Figure S6. The changes of CTC count before and after therapy, Figure S7. Cumulative Poisson distribution for cells found at 7.5 mL (orange) vs 2.5 mL (blue) sampling volume, at a known concentration in blood (given for 7.5 mL for convenience), e.g., in the top left graph, the y-value at x=3 indicates the probability of getting at most 3 cells using 2.5 mL or 7.5 mL sampling volume, Table S1. Prevalence of CTCs: comparison between the metabolic-based assay (MBA) and the CellSearch (CS), Table S2. CTC count in 26 healthy donors as detected with the metabolic-based assay (MBA), Table S3. CTCs and their correlation with therapy response as assessed by imaging.
