# Supplementary material for: Dysmetabolic Circulating Tumor Cells Are Prognostic in Metastatic Breast Cancer
Source: Cancers (Basel). 2020 Apr 19;12(4):1005. doi: 10.3390/cancers12041005 (PMC7226515; doi:10.3390/cancers12041005)
Supplement: Supplementary file 1 [file cancers-12-01005-s001.pdf]

cancers

Article

Dysmetabolic Circulating Tumor Cells Are Prognostic in Metastatic Breast Cancer

Giulia Brisotto [1,‡], Eva Biscontin [2,‡], Elisabetta Rossi [3,4], Michela Bulfoni [5], Aigars Piruska [6], Simon Spazzapan [7], Cristina Poggiana [4], Riccardo Vidotto [4], Agostino Steffan [2], Alfonso Colombatti [2], Wilhelm T. S. Huck [6], Daniela Cesselli [1], Rita Zamarchi [4,*], Matteo Turetta [2,†] and Fabio Del Ben [1,†]

Supplementary Materials

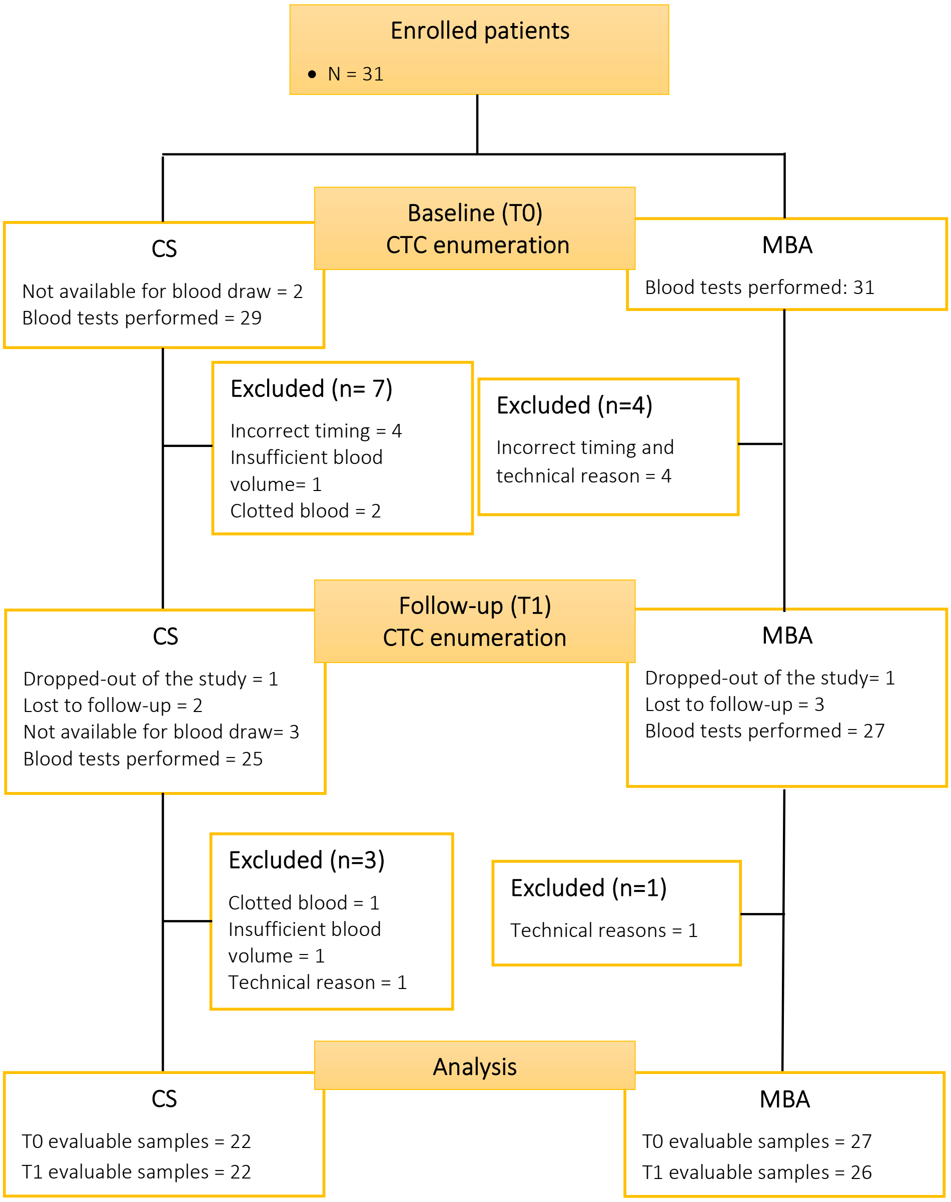

Figure S1. CONSORT diagram describing the study design, enrolment of patients and motivations of data exclusion.

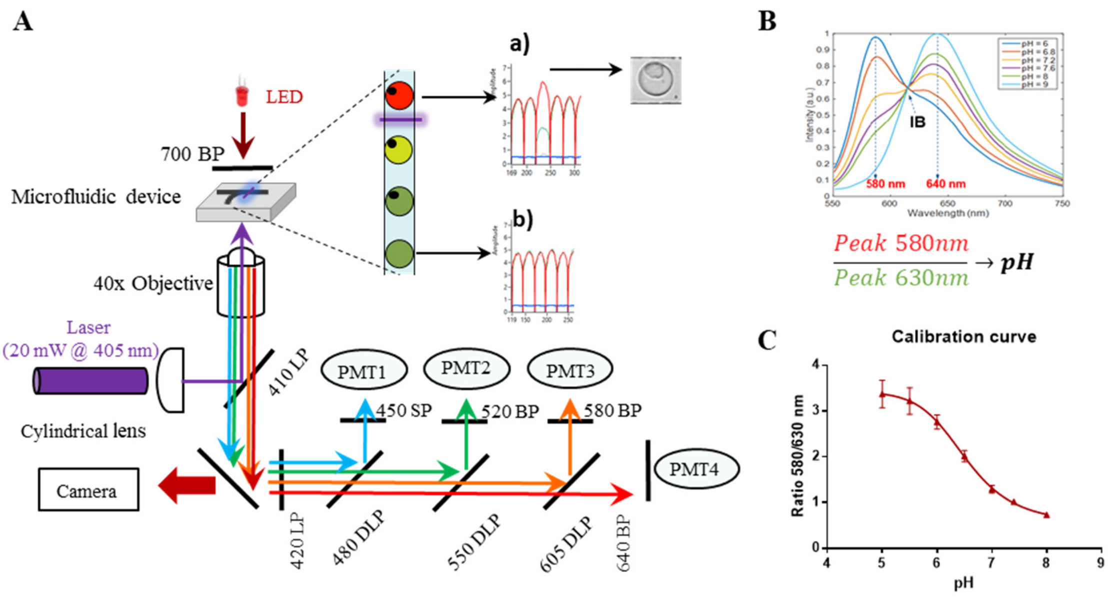

Figure S2. Principle of the metabolic-based assay (MBA) CTC detection. (**A**) Schematic overview of the optical set-up: laser light (405 nm) is shaped into a laser line through cylindrical lens and transmitted through a dichroic mirror to a 40x objective. Fluorescence light emitted from droplets was captured by the same objective, split with dichroic filters, and wavelength of interest selected by bandpass filters and measured by photomultiplier tubes (PMT) (solid line represents dichroic filters; DLP = dichroic long pass; SP = short pass). Insertions: a) representative image of an in-drop cell with the corresponding fluorescence spectrum from which a decrease of fluorescent intensity at 630nm (green line) and an increase in 580nm (red line) can be observed, as expected, from an acidic droplet; b) empty droplet showing no change in the pH, i.e. in the ratio of SNARF-5F fluorescent intensity at 580 and 630nm; (**B**) Fluorescence emission spectra of SNARF-5F showing the spectral pH-dependent shift at 580nm and 630nm. IB = Isosbestic point. As pH is lowered, SNARF-5F undergoes a wavelength shift in the emission spectrum, allowing determination of the exact pH of the droplet by measuring the fluorescence peaks at two wavelengths (ratio 580/630 nm). (**C**) Calibration curve of SNARF-5F. Ratio of 580 and 630 nm fluoresce intensity of SNARF-5F was plotted for each respective pH and a sigmoidal fit was performed to obtain the represented calibration curve.

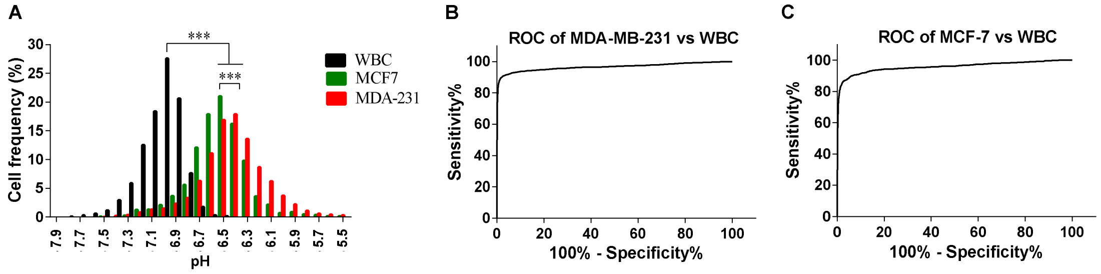

Figure 3. Measurement of ECAR by the metabolism-based assay (MBA). (**A**) Representative histogram reporting the acidification of cell-containing droplets for both breast cancer cell lines (MDA-MB-231 and MCF7) and WBCs obtained from healthy donor's sample. Both cancer cell lines had a significantly higher ECAR with respect to that detected in WBC and MDA-MB-231 reached a higher level with respect to MCF7. Statistical significance was calculated comparing cell lines and WBC by Mann-Whitney test (***p-value<0.0001). (**B**) and (**C**) ROC curves obtained comparing the ECAR of MDA-MB-231 and MCF7 breast cancer cell lines against WBC. The area under the curve was 0.96 and 0.95 in discriminating MDA-MB-231 and MCF7 from WBCs, respectively. The proportion of cells leading to a droplet acidification below pH 6.4 was 45% and 22% in the case of MDA-MB-231 and MCF7, respectively.

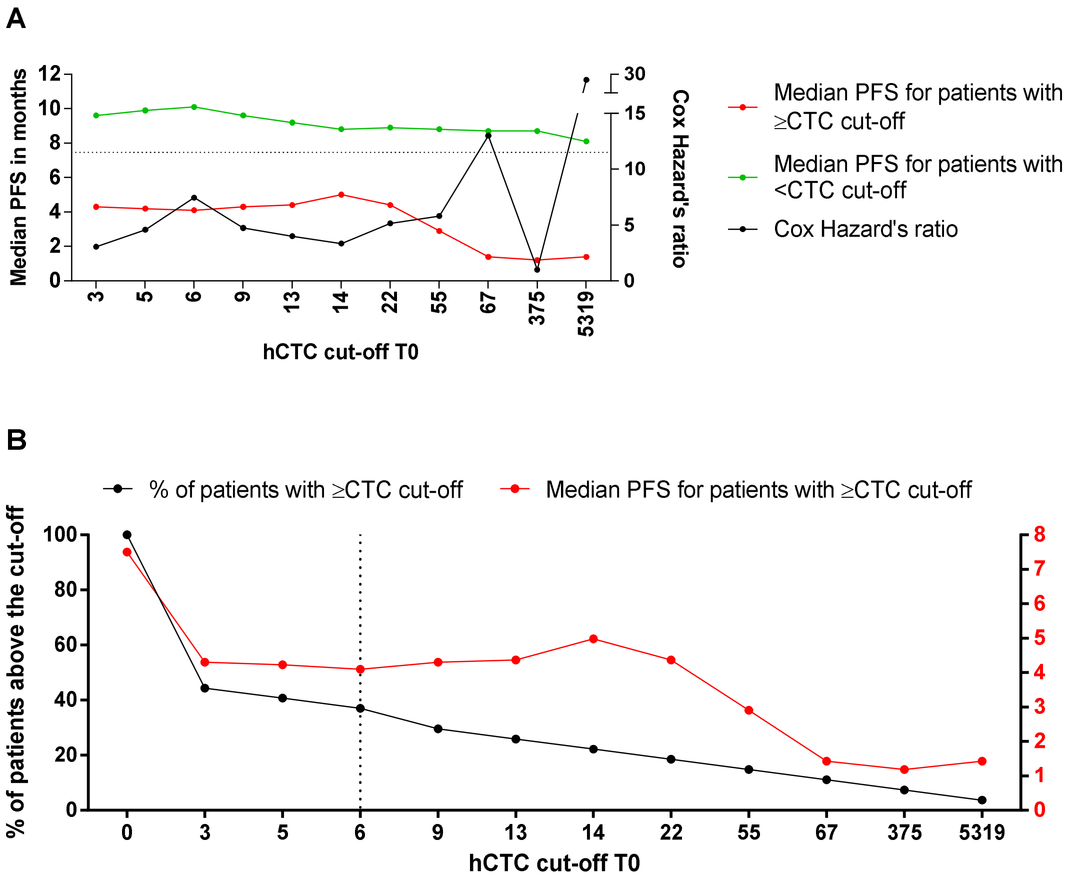

Figure S4. Determination of the prognostic CTC cut-off for the metabolic-based assay (MBA). (**A**) The median PFS for patients positive (red line) or negative (green line) and the Cox hazard ratio (black line) were plotted against each selected cut-off. (**B**) The graph reports the median PFS of positive patients and the percentage (black line) of positive patients for each selected cut-off. The dotted line represents the selected prognostic cut-off for MBA analysis. .

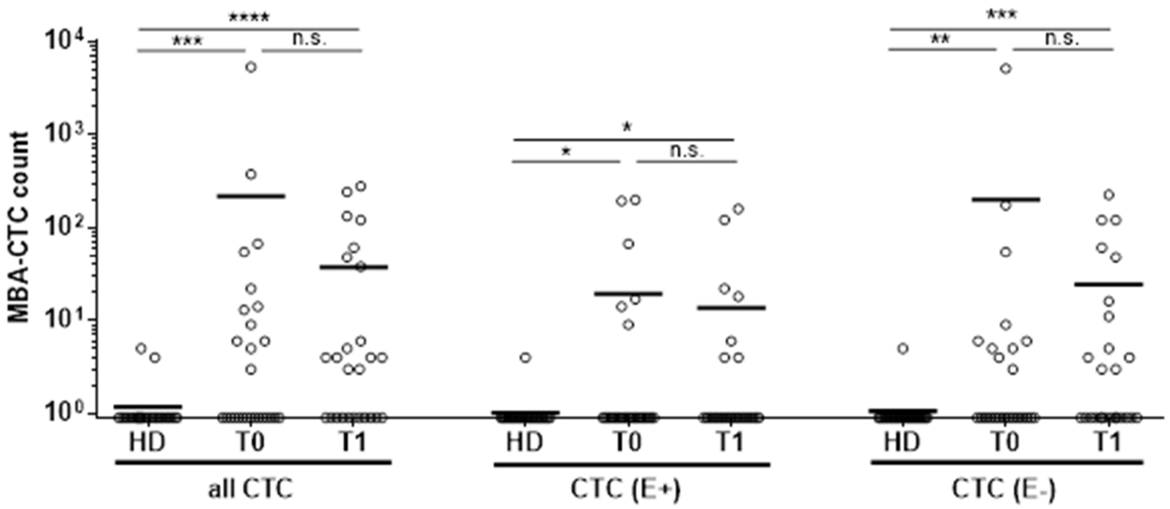

Figure S5. CTC count of healthy donors (HD) and mBC patients at baseline (T0) and follow-up (T1) performed with the metabolic-based assay (MBA). Overall, 26 healthy donors (HD), 27 patients at T0 and 26 at T1 were included for CTC enumeration. The graph reports the total CTC count (Acid-positive, i.e.: pH<6.4, droplets containing CD45-negative and both EpCAM-positive and -negative

cells) and the prevalence of the subpopulation of EpCAM-positive (E+) and EpCAM-negative (E-) CTCs. Horizontal bars represent the average CTC count. Statistical significance was evaluated by Mann-Whitney test (* p-value ≤ 0.05; *** p-value ≤ 0.001; **** p-value ≤ 0.0001).

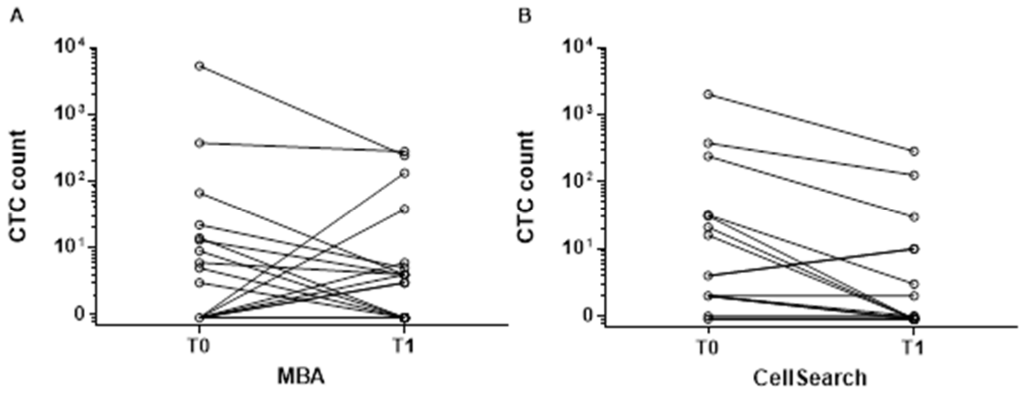

Figure S6. The changes of CTC count before and after therapy. (**A**) Trend of CTC number for each patient before (T0) and after therapy (T1) as assessed with the metabolic-based assay (MBA) or **(B)** the CellSearch. .

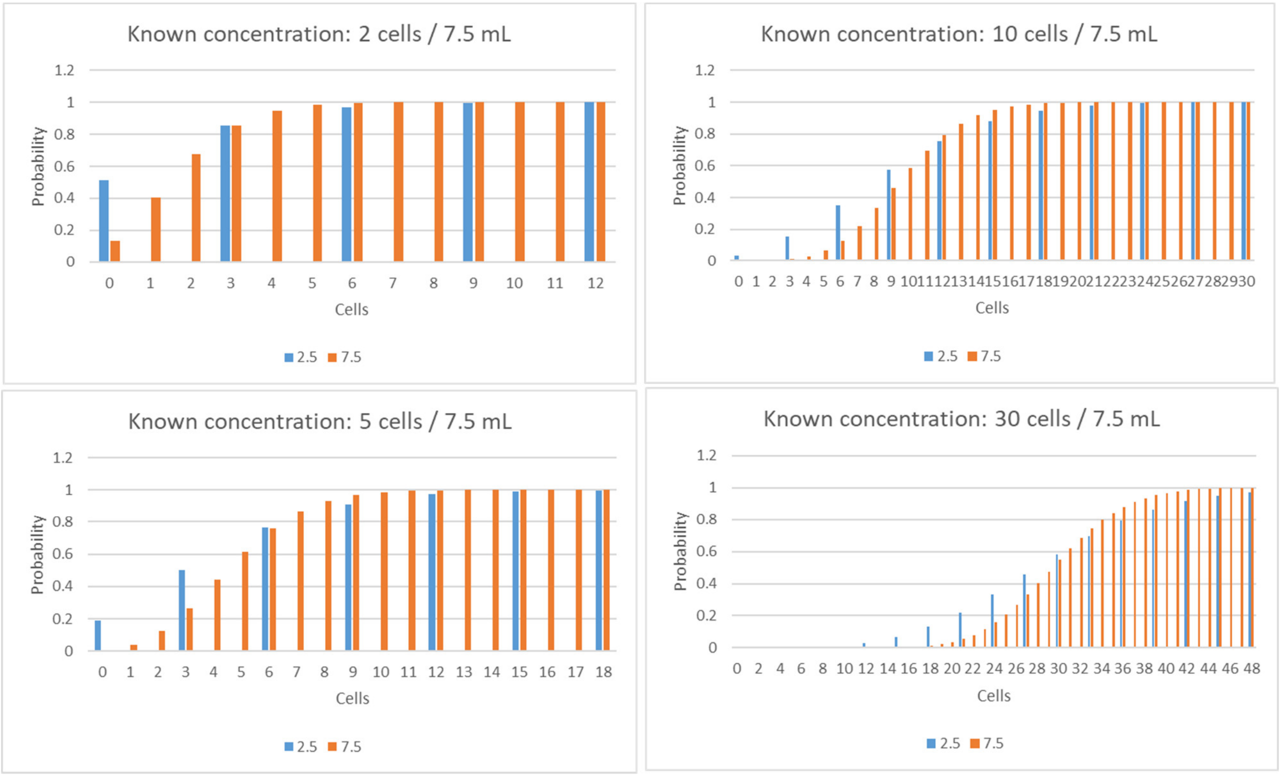

Figure S7. Cumulative Poisson distribution for cells found at 7.5 mL (orange) vs 2.5 mL (blue) sampling volume, at a known concentration in blood (given for 7.5 mL for convenience), e.g., in the top left graph, the y-value at x=3 indicates the probability of getting at most 3 cells using 2.5 mL or 7.5 mL sampling volume.

To compare the effect of sampling CTC with 7.5 mL or 2.5 mL volumes, we calculated cumulative Poisson distribution for known hypothetical concentration of CTC (corresponding roughly to 2, 5, 10 or 30 CTC/7.5 mL—266, 666, 1333 or 4000 cells per liter). In the first case—2 cells, < cut-off—we can see that the probability of detecting at most 0 cells is strongly increased in the 2.5 mL sampling volume. In other words, the probability of detecting any cell is intuitively larger with bigger sample volume. However, the probability of detecting at most 3 cells is comparable to the

sampling volume of 7.5 mL, therefore it is true that smaller sampling volume might affect the "exact" number of CTC, but to a limited extent (1–2 cells) and most importantly it is not affecting the classification of patients (> or < cut-off) because in both cases a number of cells below the cut-off will be detected.

In the second case – 5 cells, > cut-off – there is slightly increased probability of underestimating or overestimating the number of cells with smaller sampling volume (2.5 mL). However, the difference in estimation remains very limited (differing of 1–2 cells between sampling volumes). There is slightly increased probability of deeming the patient negative, but of a very little difference (the probability of detecting at most 3 cells using 2.5 mL is similar to detecting at most 4 cells using 7.5 mL. The problem is of similar entity using smaller or bigger sampling volumes.

In the third case – 10 cells, > cut-off—there is an increased probability of detecting at most 3 cells (deeming the patient negative) using 2.5 mL volume, with respect to 4 cells using 7.5 mL. The probability is 12% higher. The rest of the distribution has little difference in number of cells (differing of 3–4 cells), and will not affect classification of patients.

In the fourth case—30 cells, > cut-off—again there is increased risk of both underestimating or overestimating the exact value of CTC. However, this risk does not go over 5–6 cells of difference and most importantly will not affect the classification of patients, since there are essentially no possibilities of detecting less than 10 cells and deem the patient negative.

Table S1. Prevalence of CTCs: comparison between the metabolic-based assay (MBA) and the CellSearch (CS). The table reports patient data and the CTC count of each patient at baseline (T0) and follow-up (T1), along with i) the corresponding prevalence of EpCAM positive (E+) or negative (E− CTCs, as detected by the MBA, ii) apoptotic (M30+) CTC, as identified with the CS test; iii) first imaging response data; and iv) days of PFS and OS data locked at the time of analysis. (n.a. = not available data; see Figure S1 for details; CR=complete response; SD=stable disease; PR=partial response; PD=progressive disease).

ID	Histology	MBA						CS				1st Imaging Response (Days from T0 blood Draw)	OS	PFS
		T0			T1			T0		T1				
		CTC	CTCE+	CTCE−	CTC	CTCE+	CTCE−	CTC	CTC M30+	CTC	CTC M30+			
1	ER+/PR+/HER2-	n.a.	n.a.	n.a.	61	0	61	n.a.	n.a.	0	0	-	748	304
2	ER+/PR-/HER2-	n.a.	n.a.	n.a.	121	0	121	n.a.	n.a.	0	0	-	109	109
3	ER+/PR+/HER2-	n.a.	n.a.	n.a.	48	0	48	n.a.	n.a.	1	0	PR (111)	138	138
4	ER-/PR-/HER2-	n.a.	n.a.	n.a.	n.a.	n.a.	n.a.	n.a.	n.a.	0	0	-	714	714
5	ER-/PR-/HER2-	0	0	0	0	0	0	0	0	0	0	CR (217)	570	388
6	ER-/PR-/HER2-	375	200	175	280	160	120	379	25	127	7	PD (28)	184	28
7	ER-/PR-/HER2-	5319	194	5125	243	18	225	2022	0	288	0	PD (43)	43	43
8	ER-/PR-/HER2-	14	14	0	0	0	0	21	0	0	0	PR (153)	500	268
9	ER-/PR-/HER2-	0	0	0	6	6	0	n.a.	n.a.	n.a.	n.a.	PR (65)	499	219
10	ER-/PR-/HER2-	55	0	55	n.a.	n.a.	n.a.	0	0	n.a.	n.a.	PD (69)	208	195
11	ER+/PR-/HER2-	13	9	4	4	4	0	244	0	30	1	PD (119)	278	119
12	ER+/PR+/HER2-	6	0	6	n.a.	n.a.	n.a.	n.a.	n.a.	n.a.	n.a.	PD (82)	289	82
13	ER+/PR-/HER2-	0	0	0	4	4	0	2	0	0	0	PR (133)	484	264
14	ER+/PR+/HER2-	0	0	0	38	22	16	31	0	0	0	PD (146)	234	146
15	ER-/PR-/HER2+	0	0	0	0	0	0	n.a.	n.a.	2	0	PR (127)	478	478
16	ER+/PR+/HER2-	22	17	5	5	0	5	1	0	1	0	PD (168)	477	168
17	ER+/PR-/HER2-	0	0	0	3	0	3	4	0	10	0	PR (193)	469	360
18	ER-/PR-/HER2-	0	0	0	0	0	0	1	1	n.a.	n.a.	PR (192)	466	466
19	ER+/PR+/HER2-	0	0	0	0	0	0	2	0	1	0	PR (113)	312	260
20	ER+/PR+/HER2-	0	0	0	133	122	11	0	0	0	0	PR (62)	463	140
21	ER-/PR-/HER2+	0	0	0	0	0	0	0	0	0	0	SD (60)	462	224
22	ER+/PR-/HER2-	67	67	0	4	0	4	3	0	n.a.	n.a.	PD (131)	288	131
23	ER+/PR+/HER2-	0	0	0	n.a.	n.a.	n.a.	67	0	n.a.	n.a.	PR (181)	371	306
24	ER+/PR+/HER2-	0	0	0	3	0	3	2	0	0	0	PR (95)	364	364
25	ER+/PR+/HER2-	0	0	0	0	0	0	32	0	3	0	PD (142)	220	142
26	ER-/PR+/HER2-	3	0	3	0	0	0	n.a.	n.a.	n.a.	n.a.	-	358	358
27	ER+/PR+/HER2+	5	0	5	0	0	0	n.a.	n.a.	n.a.	n.a.	PR (67)	357	342
28	ER+/PR+/HER2-	6	0	6	4	0	4	16	0	0	0	PD (115)	164	112
29	ER+/PR-/HER2-	0	0	0	0	0	0	2	0	2	0	PR (146)	354	287

30	ER+/PR+/HER2-	9	0	9	0	0	0	4	1	10	0	PD (127)	191	127
31	ER+/PR+/HER2-	0	0	0	n.a.	n.a.	n.a.	0	0	n.a.	n.a.	PR (146)	304	304
	N	27	27	27	26	26	26	22	22	22	22			
	Mean	218	84	539	37	48	52	129	9	22	0			
	SD	1022	90	1612	75	65	70	433	14	65	1			
	Median	0	42	6	4	18	14	3	1	1	0			
	Min	0	9	3	0	4	3	0	1	0	0			
	Max	5319	200	5125	280	160	225	2022	25	288	7			

Table S2. CTC count in 26 healthy donors as detected with the metabolic-based assay (MBA). (CTC = total CTC count; CTC E+ = CTC positive for EpCAM expression; CTC E- = CTC negative for EpCAM expression).

**ID**	**MBA**		
	**CTC**	**CTC E+**	**CTC E-**
1	0	0	0
2	0	0	0
3	0	0	0
4	5	0	5
5	0	0	0
6	0	0	0
7	0	0	0
8	0	0	0
9	0	0	0
10	0	0	0
11	0	0	0
12	0	0	0
13	0	0	0
14	0	0	0
15	4	4	0
16	0	0	0
17	0	0	0
18	0	0	0
20	0	0	0
21	0	0	0
22	0	0	0
23	0	0	0
24	0	0	0
25	0	0	0
26	0	0	0
**N**	26	26	26
**Mean**	0.4	0.2	0.2
**SD**	1.3	0.8	1
**Median**	0	0	0
**Min**	0	0	0
**Max**	5	4	5

Table S3. Concordance between CTCs status and therapy response as assessed by imaging (CR = complete response; PR = partial response; SD = stable disease; PD = progressive disease; MBA = metabolism-based assay; CS = CellSearch).

**First Imaging Response**	**MBA**			**First Imaging Response**	**CS**		
	**<6**	**≥6**	***Tot***		**<5**	**≥5**	***Tot***
T0 (k=0.761)				T0 (k=0.441)			
CR/PR/SD	14	1	*15*	CR/PR/SD	10	2	*12*
PD	2	9	*11*	PD	4	6	*10*
Tot	*16*	*10*	*26*	*Tot*	*14*	*8*	*22*
T1 (k = 0.123)				T1 (k = 0.431)			
CR/PR/SD	11	3	*14*	CR/PR/SD	10	1	*11*
PD	6	3	*9*	PD	4	4	*8*
Tot	*17*	*6*	*23*	*Tot*	*14*	*5*	*19*
